# Extracellular Vesicles From TNFα Preconditioned MSCs: Effects on Immunomodulation and Bone Regeneration

**DOI:** 10.3389/fimmu.2022.878194

**Published:** 2022-05-02

**Authors:** Miya Kang, Chun-Chieh Huang, Praveen Gajendrareddy, Yu Lu, Sajjad Shirazi, Sriram Ravindran, Lyndon F. Cooper

**Affiliations:** ^1^ Department of Oral Biology, College of Dentistry, University of Illinois Chicago, Chicago, IL, United States; ^2^ Department of Periodontics, College of Dentistry, University of Illinois Chicago, Chicago, IL, United States

**Keywords:** mesenchymal stem cells, TNFα, extracellular vesicles, immunomodulation, bone regeneration

## Abstract

Mesenchymal stem cells show remarkable versatility and respond to extracellular and micro environmental cues by altering their phenotype and behavior. In this regard, the MSC’s immunomodulatory properties in tissue repair are well documented. The paracrine effects of MSCs in immunomodulation are, in part, attributable to their secreted extracellular vesicles (EVs). When MSCs migrate to the wound bed, they are exposed to a myriad of inflammatory signals. To understand their response to an inflammatory environment from an EV perspective, we sought to evaluate the effects of the inflammatory cytokine TNFα on MSC EV mediated immunomodulation. Our results indicate that while the physical characteristics of the EVs remain unchanged, the TNFα preconditioned MSC EVs possess enhanced immunomodulatory properties. *In vitro* experiments using polarized (M1 and M2) primary mouse macrophages indicated that the preconditioned MSC EVs suppressed pro-inflammatory (M1) markers such as IL-1β and iNOS and elevated reparatory (M2) markers such as Arg1 and CD206. When evaluated *in vivo* in a rat calvarial defect model, the TNFα preconditioned MSC EVs reduced inflammation at 1-, 3- and 7-days post wounding resulting in the subsequent enhanced bone formation at 4- and 8-weeks post wounding possibly by modulation of oncostatin M (OSM) expression. An analysis of EV miRNA composition revealed significant changes to anti-inflammatory miRNAs in the preconditioned MSC EVs hinting at a possible role for EV derived miRNA in the enhanced immunomodulatory activity. Overall, these results indicate that MSC exposure to inflammatory signals influence the MSC EV’s immunomodulatory function in the context of tissue repair. The specific function of TNFα preconditioned MSC EV miRNAs in immunomodulatory control of bone regeneration merits further investigation.

## Introduction

Osseous repair and regeneration following surgery or trauma begins with the inflammatory response. The importance of the inflammatory response to successful wound healing is revealed generally in studies of wound healing (1) and specifically in bone regeneration by the observed limitation in healing when effectors of inflammation are eliminated (2). Examples include the reduced healing of bone fractures in TNFα receptor knock out mice and reduced bone healing when macrophages are reduced in animal models (3, 4). Implied is the role of inflammatory cytokines in the communication between cells of the immune system and the bone forming mesenchymal cells. TNFα expression, which occurs quickly and persists for several days following injury, is a central determinant of subsequent cellular healing events (5). Mechanistically, TNFα is a mediator of MSC function that can affect the MSC proliferation and phenotype (6).

It is generally acknowledged that MSCs contribute to tissue regeneration by paracrine regulation of host cells (7, 8) resulting in immunomodulation at the site of surgery or injury. The communication and interaction of MSCs with the cells of the immune system is well documented (9). Co-culture of MSCs with dendritic cells, T-cells, and NK cells resulted in reduced inflammatory cytokine expression by all cell types. MSCs are known to direct macrophage polarization toward an M2-like (regenerative) phenotype to enhance tissue repair. The paracrine immunomodulatory function of MSCs has been attributed to the secretion of anti-inflammatory proteins and cytokines including IL-10, PGE2, NO, and TGFα.

An emerging view of immunomodulation includes the bi-directional signaling of inflammatory cells and MSCs. In the process of wound healing and tissue regeneration or repair, MSCs are recruited to an inflammatory environment. Thus, the characteristic feature of the MSC during the early stages of wound healing is one that is influenced by inflammatory cytokines with TNFα representing a dominant effector of inflammatory signaling at the site of injury of surgery. Presently, the knowledge regarding both acute and chronic TNFα exposure of MSCs suggests that acute and lower concentration TNFα signaling of undifferentiated MSCs promotes osteoinductive signaling (10, 11), while chronic and higher does MSC exposure to TNFα inhibits osteoinduction and bone formation (12, 13). The ability of the MSC to control TNFα expression by immune cells such as the macrophage may represent a key aspect of its immunomodulatory role in wound healing and in bone repair.

The paracrine signaling of MSCs is multifaceted and attributed to its production and secretion of extracellular matrix proteins, growth factors and cytokines as well as extracellular vesicles (or exosomes). Several studies indicate that many functions attributed to the MSC can be recapitulated by MSC conditioned media alone, suggesting a prominent role for the MSC secretome in control of wound healing and bone repair (14). Recent investigations have highlighted a role for MSC extracellular vesicles in the paracrine regulation of wound healing and regeneration of diverse tissues including bone (15, 16). The cargo of MSC extracellular vesicles includes protein, mRNA and microRNAs that are able to alter the function of target cells. The MSC cargo can be changed physiologically or experimentally to alter the paracrine function of the MSC. For example, overexpression of BMP2 in MSCs alters the cargo and function of secreted extracellular vesicles to enhance their osteoinductive function by directly affecting the BMP2 pathway of target cells (17).

More broadly, the concept of preconditioning of MSCs involves the exposure of MSCs to alternative culture conditions (e.g., hypoxia), to various cytokines or growth factor or through genetic modification (18). One intent of preconditioning is to increase the number of extracellular vesicles produced by the parental cell (19). Another intent is to alter the functionality of the extracellular vesicles. By example, the lineage specific differentiation of MSCs results in the production of extracellular vesicles that promote the same lineage specific differentiation of targeted MSCs (20). Other studies have demonstrated that hypoxia (21) or pro-inflammatory cytokine treatment (22) of MSCs enhanced their immunomodulatory and wound healing function. IFNγ preconditioning of MSCs also increased the anti-inflammatory protein content of MSC extracellular vesicles (23). These and other studies indicate that MSCs secrete exosomes alter immunoregulatory function during wound healing. For example, they enhance survival of allogenic skin grafts in mice in part by targeting macrophage function (24). LPS treatment of MSCs is another preconditioning strategy in which treated-MSC extracellular vesicles promotes M2 polarization (25), and attenuated inflammation in a mouse model of myocardial infraction (26).

In the present study, the treatment of MSCs with TNFα was performed to model the acute response of MSCs to inflammation following injury or surgery and to assess the effect of TNFα preconditioning of MSCs on the MSC extracellular vesicle function in bone regeneration. Comparison of TNFα treated and control MSC extracellular vesicles was performed at the structural, miRNA and functional levels both in cell culture and a calvarial model of bone regeneration. Preconditioned extracellular vesicles did not directly enhance MSC osteoinductive gene expression or differentiation in culture. However, TNFα preconditioning of MSCs resulted in the secretion of extracellular vesicles that affected macrophage polarization in cell culture and in healing calvarial tissues. Greater bone regeneration was observed in sites treated with the TNFα preconditioned MSCs compared to controls. Suggested is a bi-directional signaling of TNFα preconditioned MSCs to the macrophage that positively influences calvarial wound healing and bone regeneration.

## Materials and Methods

### Cell Culture

Human bone marrow derived mesenchymal stem cells (MSCs) were purchased from Lonza. MSCs were cultured in αMEM (Gibco) containing 20% fetal bovine serum (FBS, Gibco), 1% L-Glutamine (Gibco) and 1% antibiotic-antimycotic solution (Gibco). For MSC osteogenic differentiation induction, 100μg/ml ascorbic acid (Sigma), 10mM β-glycerophosphate (Sigma) and 10mM dexamethasone (Sigma) were supplemented in the αMEM growth medium (17).

Mouse bone marrow derived macrophages (BMMs) were isolated from 8-week-old C57BL/6J mice as per previously published protocol (27). BMMs were cultured in Dulbecco’s Modified Eagle Medium (DMEM, Gibco) containing 20% fetal bovine serum (Gibco) and 1% antibiotic-antimycotic solution (Gibco). 20ng/ml recombinant macrophage colony-stimulating factor (M-CSF, Peprotech) was supplemented into the DMEM growth medium to induce macrophage differentiation. For M1 or M2 polarization, BMMs were treated with 100ng/ml lipopolysaccharides (LPS, Sigma) with 50ng/ml Interferon gamma (IFNγ, Peprotech) or 20ng/ml Interleukin 4 (IL-4, Peprotech) for 48 hours.

### EV Isolation and Characterization

For preconditioning, MSCs were treated with 20ng/ml Tumor necrosis factor alpha (TNFα) for 72 hours. Control MSCs (no TNFα treatment) and TNFα preconditioned MSCs were washed in PBS and cultured in serum free αMEM growth medium for 24 hours. EVs were isolated from serum free culture medium according to our published and standardized protocols (20). The culture medium was harvested, cell debris were removed by centrifugation (3,000xg, 15min) and EVs were isolated using the ExoQuick-TC reagent (System Biosciences) as per the manufacturer’s recommended protocols. The isolated EVs were characterized for number and size distribution and presence of membrane markers by nano tracking analysis (NTA) and immunoblotting. For NTA, a 1/100 dilution of the EV suspension was analyzed in the Nanosight NS-300 instrument to obtain the average number of particles and the size distribution plot. Based on the NTA results, approximately equal concentration of control and TNFα EVs (1.8x10^10^ particles/ml) were used for each experiment.

For immunoblotting, exosomal proteins were isolated in RIPA buffer and 20-30 µg of EV lysate was resolved by SDS-PAGE gel, transferred onto nitrocellulose membranes and probed with primary mouse monoclonal anti-CD63 [TS63] (1/1000, ab59479, Abcam) and mouse monoclonal anti-HSP70 [C92F3A-5] (1/200, sc-66048, Santa Cruz) antibodies and near infrared dye conjugated secondary antibodies (1/15,000, Licor) as per previously published protocols (28). The blots were imaged using a Licor Odyssey imager.

### Quantitative and Qualitative Endocytosis of EVs

EVs were fluorescently labeled using the ExoGlow exosome protein labeling kit (System Biosciences) as per the manufacturer’s protocols. For quantitative experiment, MSCs and BMMs were seeded onto 96 well tissue culture plates (10,000 MSCs/well, 20,000 BMMs/well) and incubated for overnight to facilitate cell attachment. The cells were then incubated with increasing numbers of fluorescently labeled EVs for 2 hours at 37°C, washed with PBS and fixed in neutral buffered 4% paraformaldehyde (PFA). The fluorescence from the endocytosed EVs was observed and quantified by using a BioTek Synergy 2 96 well plate reader equipped with the appropriate filter sets to measure green fluorescence. The results were plotted as mean (+/− SD) relative fluorescence intensity % increase (normalized to no EV group) as a function of dosage (n=6 per group).

For qualitative endocytosis experiments, 50,000 MSCs or BMMs were seeded onto cover glasses placed in 12 well or 24 well cell culture plates. Fluorescently labeled EVs (1.8x10^9^ particles/well) were added and incubated for 2 hours. The cells were then washed with PBS, fixed in 4% PFA, permeabilized and counter stained using Alexa Fluor^®^ 568 Phalloidin (1/2000, A12390, Invitrogen) antibody. The cover glasses were then mounted using mounting medium with DAPI (Vector Laboratories) and imaged using a Zeiss LSM 710 Meta confocal microscope.

### EV Mediated MSC Osteogenic Differentiation

To examine the osteoinductive function of EVs, 50,000 MSCs were seeded onto 12 well tissue culture plates and cultured in growth medium with EVs (1.8x10^9^ particles/well) for 3 days. Total RNA was isolated using RNeasy mini kit (Qiagen) as per the manufacturer’s protocol. The RNA concentration was measured using NanoDrop One. After first strand cDNA synthesis, osteogenic related gene specific primers (**Table 1**) were used to direct PCR amplification and SYBR Green probe incorporation using a BioRad CFX96 thermocycler. All expression data were normalized to housekeeping genes GAPDH and fold change was calculated using ΔΔCt method (n=4 per group).

**Table 1:** Primer pairs used for qRT-PCR.

Genes	Forward (5’-3’)	Reverse (5’-3’)
mouse GAPDH	AGGTCGGTGTGAACGGATTTG	GGGGTCGTTGATGGCAACA
mouse IL-1β	GCAACTGTTCCTGAACTCAACT	ATCTTTTGGGGTCCGTCAACT
mouse iNOS	GTTCTCAGCCCAACAATACAAGA	GTGGACGGGTCGATGTCAC
mouse TNFα	CAGGCGGTGCCTATGTCTC	CGATCACCCCGAAGTTCAGTAG
mouse CD206	CTCTGTTCAGCTATTGGACGC	CGGAATTTCTGGGATTCAGCTTC
mouse Arg1	CTCCAAGCCAAAGTCCTTAGAG	AGGAGCTGTCATTAGGGACATC
mouse IL-10	GCTCTTACTGACTGGCATGAG	CGCAGCTCTAGGAGCATGTG
mouse OSM	ATGCAGACACGGCTTCTAAGA	TTGGAGCAGCCACGATTGG
human GAPDH	CAGGGCTGCTTTTAACTCTGG	TGGGTGGAATCATATTGGAACA
human BMP2	ACTACCAGAAACGAGTGGGAA	GCATCTGTTCTCGGAAAACCT
human RUNX2	TGGTTACTGTCATGGCGGGTA	TCTCAGATCGTTGAACCTTGCTA
human OSX	CCTCTGCGGGACTCAACAAC	AGCCCATTAGTGCTTGTAAAGG
rat IL-1β	CACCTCTCAAGCAGAGCACAG	GGGTTCCATGGTGAAGTCAAC
rat TNFα	AAATGGGCTCCCTCTCATCAGTTC	TCTGCTTGGTGGTTTGCTACGAC

For *in vitro* differentiation, MSCs were differentiated as described above using osteogenic differentiation medium. For the alkaline phosphatase (ALP) assays, MSCs (50,000 cells/well) were seeded onto 12 well tissue culture plates and EVs (1.8x10^9^ particles/well) were added to the cells and cultured up to 7 days. ALP activity was quantified using Alkaline phosphatase assay kit (Abcam) by measuring p- nitrophenyl (pNP) based on the spectrophotometric absorbance at 405nm. The fold change of ALP activity at each time point was calculated with respect to relative enzymatic activity of day 0 (n=4 per group). To observe the calcium deposition, alizarin red staining was performed. MSCs (100,000 cells/well) were seeded onto 6 well tissue culture plates and EVs (3.6x10^9^ particles/well) were added to the cells and incubated for 14 days. The cells were then washed with PBS, fixed with 4% PFA and stained with Alizarin red solution (Sigma).

### EV Mediated BMM Polarization

The functionality of EVs on BMM polarization was assessed by qRT-PCR and MILLIPLEX multiplex assay (Millipore Sigma). Briefly, 250,000 BMMs were seeded onto 24 well tissue culture plates and incubated for overnight prior to the treatment. The cells were polarized to M1 or M2 phenotype as described in cell culture section and EVs (4.5x10^9^ particles/well) were added to the cells and cultured for 48 hours. For multiplex assay, the culture medium was harvested, and cell debris were removed by centrifugation (3,000g, 15min). The protein concentration was measured using Pierce BCA protein assay kit (Thermo Fisher) and culture medium was adjusted to have equal amount of protein per well (n=4 per group). The multiplex assay was performed as per manufacture’s recommended protocols and the concentration of macrophage secreted protein was quantified using Magpix system at UIC RRC Flow cytometry core. For qRT-PCR, the total RNA from BMMs were isolated using RNeasy mini kit (Qiagen) as per the manufacturer’s protocol and the concentration of RNA was measured by NanoDrop One. Equal amount of RNA was used to complete first strand cDNA synthesis and macrophage polarization related gene specific primers (**Table 1**) were used to performed qRT-PCR. All expression data were normalized to housekeeping genes GAPDH and fold change was calculated using ΔΔCt method (n=4 per group).

### Rat Calvaria Defects

To evaluate the effects of EVs on bone healing, the rat calvaria defect model was used. The rats were anesthetized intraperitoneally using Ketamine (80mg/kg)/Xylazine (10mg/kg) and a vertical incision was made in the head at the midline to expose the calvarial bone. Two 5mm calvarial defects were created bilaterally in the calvarium without dura perforation using a trephine burr. A clinical grade collagen scaffold (OraPLUG, Salvin) was placed on the wound with PBS (control), control EVs or TNFα EVs (4.5x10^9^ particles/defect). The rats were sacrificed by carbon dioxide asphyxiation followed by cervical dislocation at each timepoint. For early time points, the embedded scaffolds were harvested and subjected to histology and qRT-PCR at day 1, 3 and 7 post-surgeries. Total RNA was isolated using miRNeasy mini Kit (Qiagen) as per the manufacturer’s protocol and qRT-PCR was performed as described in the previous section using rat specific primers (**Table 1**). The fold change of expression level was calculated using ΔCt method (n=4 per group). At 4- and 8-weeks post-surgery, the calvaria were harvested, fixed in neutral buffered 4% paraformaldehyde and subjected to 3D μCT analysis using a Scanco40 μCT scanner. The data obtained from the μCT scanner was quantitatively analyzed using a custom-built MATLAB Program.

### Histology and Immunohistochemistry

After 3D μCT analysis, the calvaria samples were decalcified in 10% EDTA solution. The harvested scaffolds at day 1, 3, and 7 and the decalcified calvaria at 4- and 8- weeks were then embedded in paraffin and sectioned into 5-10μm sections. The Hematoxylin and eosin (H&E) staining was performed as per previously published protocols (28). For immunofluorescent staining, the slides were pre-treated with 5% BSA blocking buffer for an hour at room temperature. Macrophage markers were stained with rabbit polyclonal anti-inducible nitric oxide synthase (iNOS) antibody (1/100, ab15323, Abcam), rabbit monoclonal [D4E3M] anti-arginase 1 (Arg1) antibody (1/100, 93668, Cell signaling), and mouse monoclonal [A-9] anti-oncostatin m (OSM) antibody (1/100, sc-374039, Santa Cruz). Osteomarkers were stained with mouse monoclonal [65529.111] anti-bone morphogenetic protein 2 (BMP2) antibody (1/100, ab6285, Abcam), mouse monoclonal [LFMb-31] anti-DMP-1 antibody (1/100, sc-73633, Santa Cruz), and mouse monoclonal [LFMb-25] anti-BSPII antibody (1/100, sc-73630, Santa Cruz). Sections were then stained with anti-mouse FITC and anti-rabbit TRITC secondary antibodies (1/200, Sigma) and imaged using Zeiss LSM 710 laser scanning confocal microscope. ImageJ was used to quantify the immunostained number or %area per field (n=4 per group). The positive number of iNOS and Arg 1 cells was normalized to the number of nuclei per field.

### MicroRNA Sequencing

To study the microRNA (miRNA) profiles of TNFα EVs, miRNA sequencing was performed. Total RNA in EVs was extracted using miRNeasy mini Kit (Qiagen). Next-generation sequencing (NGS) was performed for miRNA expression profile. One microgram of total RNA was used for the construction of cDNA library using the TruSeq Small RNA sample prep kits (Illumina). Sequencing was performed with Illumina NovaSeq 6000 (LC Sciences). A proprietary analysis pipeline by LC Sciences, ACGT101-miR v4.2, was used for data analysis, where reads were mapped to the human genome (GRCh38) and miRBase (https://mirbase.org, Release 22.1). The normalization method of the data was to divide the sequence counts of individual samples by the corresponding normalization factors, which were the median of the ratios of specific sample counts to geometric mean counts of all samples. Student’s t-test (two-tailed, n=3) was used to analyze expression differences between TNFα EV and control EV group, and the differentially expressed miRNAs were showed in the heatmap.

Kyoto Encyclopedia of Genes and Genomes (KEGG) pathway analysis was performed using ACGT101-miR v4.2 to evaluate the functions of differentially expressed miRNA targets and gene interactions. Significance was determined by performing Fisher’s exact test (P< 0.05).

### Statistical Analysis

For experiments involving two groups, student’s t-test with a confidence interval of 95% was utilized. For the experiments involving comparison of more than two groups, One-way ANOVA was performed with a confidence interval of 95%, following by pairwise comparisons using Tukey’s *ad-hoc* method (P<0.05).

## Results

### Effect of TNFα Treatment on MSC EV Properties and EV Characterization

**Figure 1:**
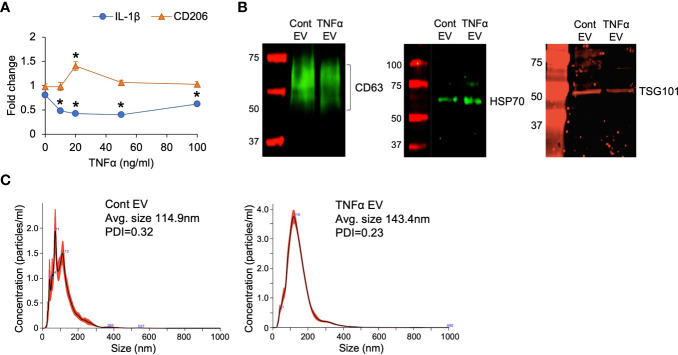
EV characterization. **(A)** The effect of TNFα preconditioned MSC EVs on the expression of IL-1β and CD206 in M1 polarized primary macrophages. Data is presented for two genes (IL-1β and CD206) as representatives of M1 and M2 markers respectively. Fold change was calculated with respect to M1 macrophages in the absence of EVs. X-axis points refer to different concentrations of TNFα used for preconditioning. 20ng/ml was chosen as an ideal concentration for the subsequent experiments. *: statistical significance (P < 0.05) with respect to no EV control by Student’s t-test. **(B)** Expression of EV markers CD63, HSP70 and TSG101 in naïve (control) and TNFα EVs. **(C)** NTA plots of control and TNFα EVs showing their size distribution.

To observe if preconditioning of MSCs by treatment with TNFα affects the anti-inflammatory property of MSC EVs, MSCs were treated with varying concentrations of TNFα. The anti-inflammatory effects of the derivative EVs were analyzed by evaluating the expression levels of IL-1β (a pro-inflammatory cytokine) and CD206 (a marker for M2 polarized macrophage) in M1 polarized BMMs. Results presented in **Figure 1A** indicate that TNFα preconditioning enhanced the anti-inflammatory property of MSC EVs by reducing IL-1β expression of M1 polarized BMMs at 10ng/ml and enhancing the expression of CD206 at 20ng/ml. Based on the results, 20ng/ml concentration was determined to be the ideal concentration for preconditioning and this concentration was used in subsequent experiments to generate TNFα preconditioned MSC EVs (TNFα EVs).

The properties of the TNFα EVs were analyzed in comparison to naïve MSC EVs by immunoblotting for EV specific markers CD63, HSP70 and TSG101 as well as by nano particle tracking analysis (NTA) to obtain the EV size distribution. Results presented in **Figures 1B** shows the similarity in the expression levels of the selected EV markers. NTA (**Figure 1C**) showed a minor increase in the average particle size of the TNFα EVs although this was not statistically significant. Similar polydispersity index between control and TNFα EVs was observed. The EVs from TNFα treated MSCs showed similar expression of EV markers as well as size distribution and PDI. Overall, these results indicated that treatment of MSC with TNFα did not cause global changes in structure or measured biochemistry of MSC EVs.

### Effects of TNFα EVs on Osteogenic Differentiation of MSCs

**Figure 2:**
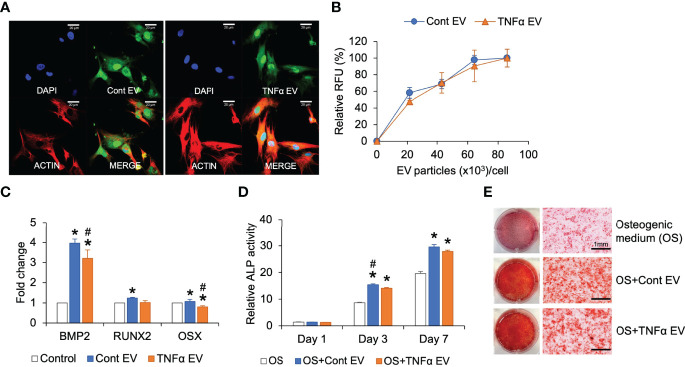
Effect of TNFα EVs on MSC osteogenic differentiation. **(A)** Representative confocal images of fluorescently labeled control and TNFα EVs (green) endocytosed by MSCs. Scale bar=20μm in all images. The red staining in the images indicate actin counter stain and blue staining shows the nuclei (DAPI). **(B)** Dose-dependent and saturable endocytosis of fluorescently labeled control and TNFα EVs by MSCs. **(C)** Fold change in the expression of osteoinductive marker genes in MSCs in the presence of control and TNFα EVs (72hrs post treatment) in the presence of growth medium. Data represents fold change with respect to no EV controls. Note that TNFα preconditioning does not generate osteoinductive property in MSC EVs. **(D)** ALP activity in MSCs cultured in osteogenic differentiation medium in the presence/absence of control or TNFα EVs. Note that while EVs increase ALP activity in general, TNFα preconditioning does not increase osteoinductive potential. **(E)** Representative images of alizarin red stained MSC cultures after 14 days of culture in osteogenic medium in the presence/absence of EVs. Note that while EV presence increased calcium deposition, no change was observed between control and TNFα preconditioned groups. *: statistical significance (P < 0.05) with respect to untreated control, #: statistical significance (P < 0.05) between control and TNFα EV groups as calculated by Tukey’s test post ANOVA.

MSC EVs influence osteogenic differentiation of MSCs. To evaluate if preconditioning of MSCs with TNFα further influences the osteoinductive ability of the derivative EVs, we first analyzed the ability of control and TNFα EVs to be endocytosed by MSCs. Congruent with their biophysical similarity, the results presented in **Figures 2A, B** show qualitatively and quantitatively that no significant differences were observed in the endocytic properties of the two types of EVs. Both were effectively endocytosed. When MSCs were treated with control or TNFα EVs in the presence of growth medium, both EVs triggered a positive change in the expression level of osteogenic marker BMP2 with TNFα EVs less effective than control EVs. The changes effected by both types of EVs were statistically significant (P<0.05, Tukey’s test post ANOVA) with respect to untreated controls (* in **Figure 2C**). Little change was observed in the expression levels of RUNX2 and osterix (OSX) transcription factors. We also evaluated alkaline phosphatase (ALP) activity in MSCs under the influence of osteogenic differentiation medium in the presence/absence of control and TNFα EVs. Results presented in **Figure 2D** show that both EVs enhance ALP activity. However, the TNFα EVs were less effective than control EVs with the day 3 data point being statistically significant (P<0.05, Tukey’s test post ANOVA) and the day 7 results not statistically significant while showing a minor reduction with the TNFα EVs. Concurrently, MSCs cultured under osteogenic differentiation medium for 14 days in the presence/absence of control and TNFα EVs were stained with alizarin red to evaluate calcium deposition. While both EVs enhanced calcium deposition with respect to control, no differences were observed among the two types of EVs (**Figure 2E**). Overall, these experiments indicated that preconditioning of MSCs with TNFα did not significantly alter the osteoinductive properties of MSC EVs.

### Effects of TNFα EVs on Macrophage Polarization

**Figure 3:**
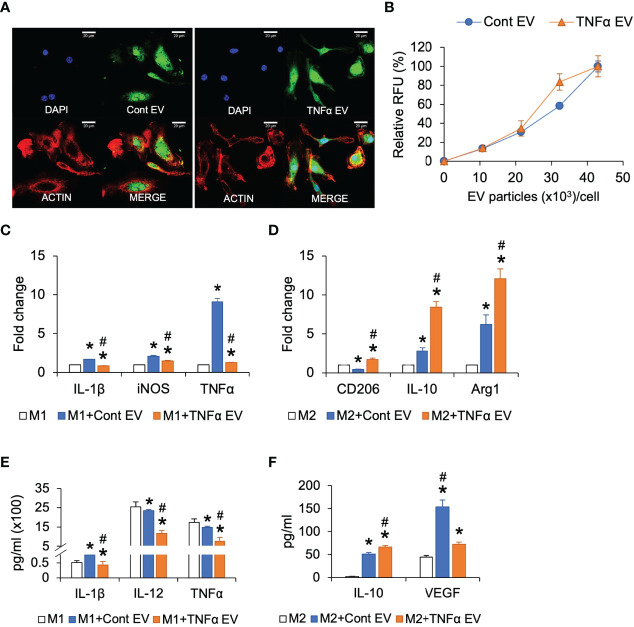
Effect of TNFα EVs on macrophage polarization. **(A)** Representative confocal images of fluorescently labeled control and TNFα EVs (green) endocytosed by macrophages. Scale bar=20μm in all images. The red staining in the images indicate actin counter stain and blue staining shows the nuclei (DAPI). **(B)** Dose-dependent endocytosis of fluorescently labeled control and TNFα EVs by primary macrophages. **(C)** Fold change in gene expression of pro-inflammatory markers in M1 polarized macrophages in the presence/absence of EVs. Note that TNFα EVs significantly reduce the expression levels of IL-1β, iNOS and TNFα indicating a reduction in M1 polarization. **(D)** Fold change in the expression levels of M2 marker genes in M2 polarized macrophages in the presence/absence of EVs. Note the significant increase in the expression levels of M2 markers CD206, IL-10 and Arg1 indicating an increase in M2 polarization. **(E)** Multiplex ELISA determined quantitative expression of pro-inflammatory cytokines in M1 polarized macrophages in the presence/absence of EVs. Note the significant reduction in cytokine expression in the presence of TNFα preconditioned MSC EVs. **(F)** Multiplex ELISA determined quantitative expression of IL-10 and VEGF in M2 polarized macrophages. *: statistical significance (P < 0.05) with respect to untreated control, #: represents statistical significance (P < 0.05) between control and TNFα EV groups as calculated by Tukey’s test post ANOVA.

Results presented in **Figure 1A** showed that preconditioning MSCs with TNFα altered the immunomodulatory effects of the MSC EVs. To characterize this further, we evaluated the effects of TNFα EVs on polarized (M1 and M2) BMMs in comparison to control EVs. As in the case of MSCs, we first evaluated the ability of control and TNFα EVs to be endocytosed by BMMs. Results presented in **Figures 3A, B** indicate that there is no significant difference between the two types of EVs with respect to endocytic properties. However, when M1 polarized macrophages were treated with control or TNFα EVs, the TNFα EVs had the anti-inflammatory effect of reducing the gene expression levels of IL-1β, iNOS and TNFα significantly compared to control EVs (**Figure 3C**). The protein expression levels of IL-1β, IL-12 and TNFα was also measured by multiplex ELISA for this experiment. Results presented in **Figure 3E** show that the TNFα EVs significantly reduced the expression levels of all three inflammatory cytokines with respect to controls (*) as well as control EVs (#).

BMMs were alternatively polarized to M2 phenotype and subjected to treatment with the control or TNFα EVs. Results presented in **Figure 3D** show that the TNFα EVs significantly enhanced the gene expression levels of M2 markers CD206, IL-10 and Arg1 with respect to both untreated controls (*) as well as control EVs (#). Protein expression levels were measured by multiplex ELISA and results presented in **Figure 3F** show that the anti-inflammatory cytokine IL-10 expression was significantly increased in TNFα EV group compared to control (*) and control groups (#). VEGF levels were significantly reduced with respect to control EV group although they remained at higher levels with respect to untreated controls.

Overall, the results from these studies showed that preconditioning of MSCs with TNFα did not affect the ability of the derivative EVs to be endocytosed by macrophages. However, when compared to control EVs treatment of polarized macrophages, TNFα EVs treatment caused greater reduction in pro-inflammatory M1 markers and increased expression of anti-inflammatory and reparative M2 markers.

### Immunomodulatory Effects of TNFα EVs *In Vivo*

**Figure 4:**
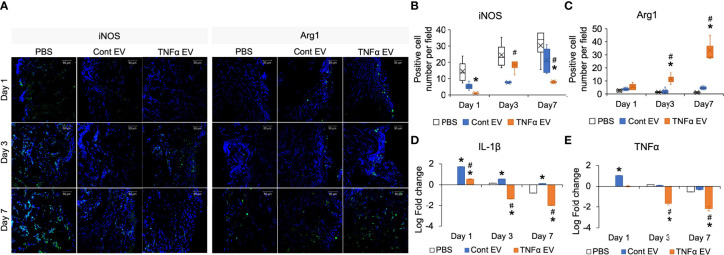
Modulation of *in vivo* inflammatory response by TNFα EVs. **(A)** Representative confocal images showing IHC staining for pro-inflammatory (M1) marker iNOS (green) and anti-inflammatory (M2) marker Arg1 (green) in tissue sections from rat calvarial defects 1-, 3- and 7-days post wounding. Scale bar=50μm in all images. Note the reduced presence of iNOS positive and increased presence of Arg1 positive cells in the TNFα EV group compared to controls. **(B, C)** Image J based quantitation of number of positive cells per field of view for iNOS and Arg1 expression (n = 5). Note the significant reduction in iNOS positive cells and increase in Arg1 positive cells. **(D, E)** Gene expression of IL-1β and TNFα respectively in tissue samples of calvarial wounds 1, 3 and 7 days post wounding. Data are represented as log 10 of fold change. Note the significant drop in the expression of these pro-inflammatory cytokines in the presence of TNFα EVs with respect to controls. *: statistical significance (P < 0.05) with respect to untreated control, #: statistical significance (P < 0.05) between control and TNFα EV groups as calculated by Tukey’s test post ANOVA.

Results presented in **Figure 3** indicated that TNFα preconditioning can reduce pro-inflammatory activity and enhance the anti-inflammatory/reparative activity of macrophages. We extended these *in vitro* observations by evaluating the ability of control and TNFα EVs to influence inflammation occurring *in vivo* in a rat calvarial defect model. For these experiments, we chose to evaluate the effects of the EVs at days 1, 3 and 7 post wounding qualitatively and quantitatively. The wounds were treated with collagen membranes containing the respective EVs or PBS and samples were harvested at days 1, 3 and 7 post wounding. Paraffin embedded samples were immunoassayed for iNOS as a pro-inflammatory marker (M1-like) and Arg1 as an anti-inflammatory marker (M2-like). Results presented in **Figure 4A** indicate a reduced number of iNOS positive cells in the TNFα EV group compared to untreated (PBS) and control EV group. Quantitation of number of cells per field of view across 5 samples is presented in **Figures 4B, C**. The results validate the *in vitro* observations and show a clear (30-90%) reduction in iNOS positive cells and an increase (>200%) in Arg1 positive cells over the treatment period. The gene expression levels of IL-1β and TNFα were also evaluated by qRT-PCR from the samples (n=4). Results are presented as log 10 fold change over untreated control at day 1 for both genes. Expression levels depicted in **Figures 4D, E** show a robust reduction in the expression levels of IL-1β and TNFα in TNFα EV group compared to both untreated controls (*) as well as naïve MSC EV group (#).

**Figure 5:**
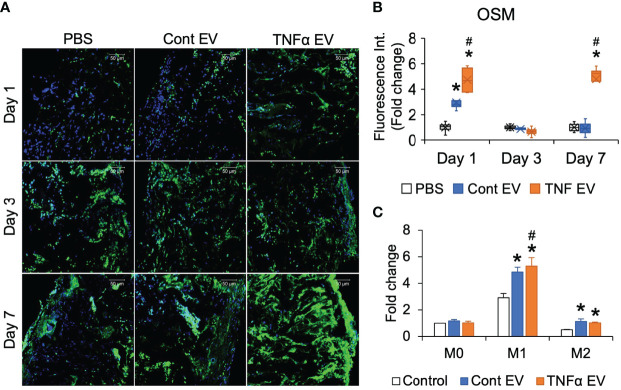
OSM expression is influenced by TNFα EVs. **(A)** Representative confocal micrographs of 1-, 3- and 7-day calvarial sections immunoassayed for OSM (green). Scale bar=50μm in all images. **(B)** Image J based quantitation of fluorescence intensity in tissue sections (n = 5). Data are presented as fold change in intensity with respect to untreated (PBS) controls. Note the significant increase in OSM intensity in the TNFα EV group compared to controls. *: statistical significance (P < 0.05) with respect to untreated control, #: statistical significance (P < 0.05) between control and TNFα EV groups as calculated by Tukey’s test post ANOVA. **(C)** Fold change in OSM expression in primary macrophages under naïve (M0), M1 and M2 polarization conditions in the presence/absence of control and TNFα EVs. Data are presented as fold change over M0 control (no EV) OSM expression. *: statistical significance (P < 0.05) with respect to no EV control in each polarization condition, #: statistical significance (P < 0.05) between control and TNFα EV groups as calculated by Tukey’s test post ANOVA.

Published studies have shown the relationship between inflammation and bone regeneration and the effects of the secretome of macrophages on MSC differentiation and osteoinduction (27, 28). One of the factors secreted by macrophages that positively influences bone repair is oncostatin M (OSM) (29). Therefore, to evaluate if, apart from controlling the expression of inflammatory markers, TNFα EVs also influence the expression of OSM, we quantified OSM expression by immunoassay of days 1, 3 and 7 sections for OSM. Results presented in **Figure 5A, B** show that TNFα EVs triggered a robust increase in the expression levels of OSM *in vivo.* This was also verified in *in vitro* experiments in macrophages polarized to M0, M1 and M2 phenotypes in the presence/absence of control or TNFα EVs (**Figure 5C**).

Overall, these *in vivo* results corroborated our *in vitro* observations and show that TNFα preconditioning enhances the positive immunomodulatory effects of MSC EVs by reducing the expression of pro-inflammatory markers and enhancing the expression of reparative anti-inflammatory markers.

### Effect of TNFα EVs on Bone Repair *In Vivo*

**Figure 6:**
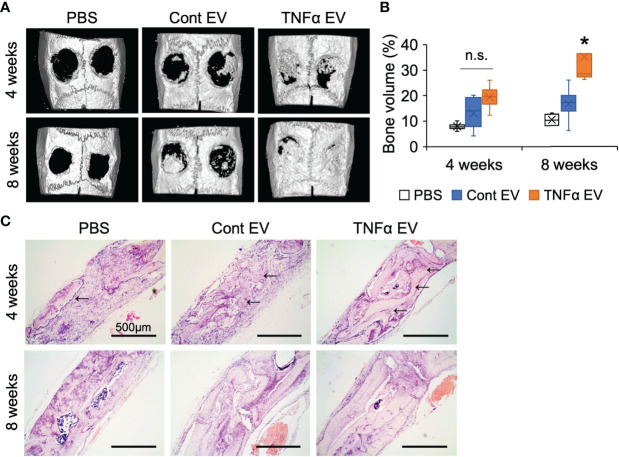
Bone regeneration is influenced by TNFα EVs. **(A)** Representative 3D μCT images of rat calvarial defects 4- and 8-weeks post wounding. Note the increased presence of bone in EV groups compared to control and the increased bone volume in the TNFα EV group compared to the other two. **(B)** volumetric quantitation of regenerated bone represented as percentage of BV/TV. Note the significant increase in bone volume at 8 weeks in the TNFα EV group compared to controls. n.s, Not significant;*: statistical significance (P < 0.05) with respect to PBS and control EV group as calculated by Tukey’s test post ANOVA. **(C)** Representative micrographs of calvarial defect sections stained with H&E. Black arrows point to newly formed bone in the defect. Scale bar=500μm.

**Figure 7:**
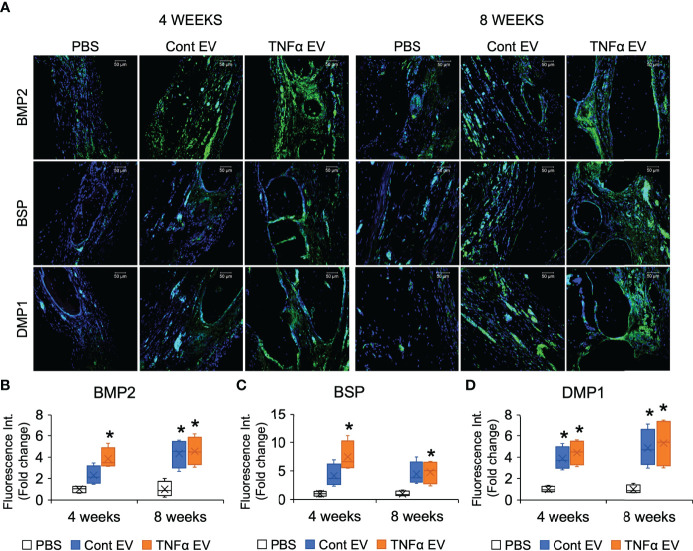
Expression of osteogenic markers in rat calvarial defects. **(A)** Representative confocal micrographs representing the expression of osteogenic markers BMP2 (green), BSP (green) and DMP1 (green) in decalcified calvarial sections 4- and 8- weeks post wounding. Scale bar=50μm in all sections. **(B–D)** are Image J based quantitation of fluorescence intensity of BMP2, BSP and DMP1 in the sections (n = 5) represented as fold change with respect to untreated (PBS) control. *: statistical significance (P < 0.05) with respect to PBS controls calculated by Tukey’s test post ANOVA. Note that while the EV treated groups showed increased expression compared to PBS control, no significant difference was observed in expression intensity between control and TNFα EVs.

To evaluate if the immunomodulatory effects observed at the shorter time points translated to increased bone repair at later time points, we evaluated the effects of the two types of EVs in the calvarial defect model after 4- and 8-weeks post wounding. Quantitative μCT analysis of the calvarial samples indicated that TNFα EVs enhanced bone repair at 4 and 8 weeks (greater than 2-fold at 8 weeks; **Figures 6A, B**). Confirmatory histology of the decalcified samples revealed increased bone formation in the TNFα EV group at both time points (**Figure 6C**). The sections were also immunoassayed for the expression of BMP2, bone sialoprotein (BSP) and dentin matrix protein 1 (DMP1). Results presented in **Figure 7A** and quantified in **Figures 7B–D** indicate that while both control and TNFα EVs both triggered an increase in the expression of all three osteoinductive proteins, no significant differences were observed in expression levels between the two groups. Overall, these results indicate that the immunomodulatory effects of TNFα EVs translate to enhanced healing of calvarial wounds in wild type rats.

### MiRNA Composition of TNFα EVs

**Figure 8:**
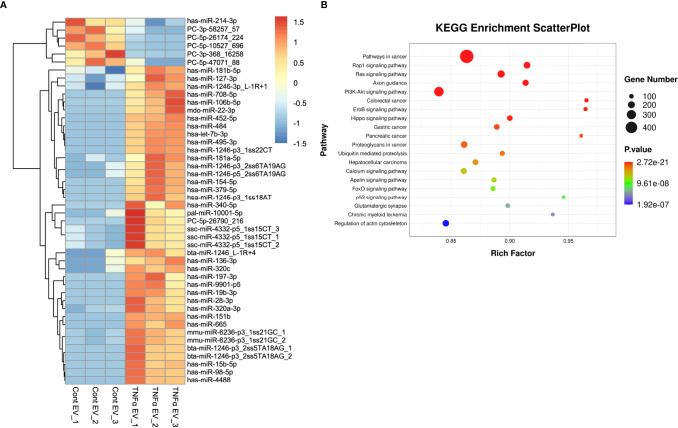
miRNA components in TNFα EVs. **(A)** A heat map of the differentially expressed miRNAs in control and TNFα EVs (n = 3). **(B)** KEGG analysis of relevant pathways significantly affected by differentially expressed miRNAs in TNFα EVs. Statistical significance (P < 0.05) was determined by performing Fisher’s exact test.

In prior studies, MSC EV functionality has been directly linked to its miRNA composition (30). We sequenced both control and TNFα EVs to identify differentially expressed miRNAs that might contribute towards the EVs altered functionality. The heat map presented in **Figure 8A** shows the differentially regulated miRNAs in the TNFα EVs compared to control EVs. The corresponding KEGG analysis highlights that the change in miRNA composition accounts for multiple pathways that involve immunomodulation, cancer biology, cell cycle and cell survival (**Figure 8B**).

## Discussion

Tissue engineering of bone using MSCs has its foundations based on the observations that MSCs give rise to osteoprogenitor cells (31) and their transplantation into large segmental defects supports bone regeneration (32). The mechanisms affecting MSC-mediated bone regeneration include the role of the MSC secretome in paracrine regulation of bone regeneration, particularly with respect to immunomodulatory function. Among the included secretome elements, exosomes or EVs have emerged as alone being able to reproduce many of the MSC-mediated effects in tissue regeneration (33). MSC EVs are emerging as potential regenerative tools in tissue engineering (34, 35).

MSC EVs have been used in many tissues for regeneration with varying levels of success. This generalized promotion of regeneration may reflect a more central role of the MSC in immunomodulation (9). In fact, MSCs role in immunomodulation is widely recognized and the MSC EV are able to influence immune cell function (36). More notably, MSCs and their EVs are able to influence macrophage polarization, a phenomenon well known as a central mediator of wound healing and regeneration (37).

The process of wound healing and regeneration involves the establishment of an early tissue comprised of an immature extracellular matrix containing abundant immune cells including macrophages. These macrophages appear as pro-inflammatory (M1-like) cells that function to direct the important inflammatory steps in early wound healing. TNFα is among the pro-inflammatory cytokines expressed early in the process by macrophages. It is in this environment that MSCs are recruited to sites for wound healing and regeneration. Concomitant with MSCs populating this tissue, macrophage polarization pivots toward a regenerative (M2) phenotype. The MSC is able to affect this immunomodulatory macrophage polarization switch in cell culture (28) and we proposed that MSC exposure to TNFα supports the MSC immunomodulatory function.

The present study demonstrates that TNFα preconditioning of MSCs alters the immunomodulatory effects of MSC EVs on primary macrophages. This is consistent with other studies that have demonstrated that MSC EVs are able to influence macrophage polarization [reviewed in (37)] and that TNFα treatment of gingival MSCs resulted in greater anti-inflammatory effects and a reduction in periodontal bone loss in the mouse model (38). Here, despite present cell culture studies that indicated TNFα EVs did not enhance osteoinduction in MSC cultures compare to control EVs, TNFα EVs treatment of calvaria defects significantly increased bone formation at 4 and 8 weeks. This increased bone formation was associated with the reduction in M1 macrophages and increase in M2-like macrophages in these treated tissues during the pro-inflammatory, formative period of 3 to 7 days during regeneration. A recent study demonstrated that the treatment of calvaria defects with M2 macrophage EVs promoted bone regeneration to greater extent than naïve and M1 macrophage EVs (28).

Preconditioning has been examined in the present study at the level of miRNA cargo of MSC EVs. Importantly, preconditioning has been shown to alter the primary metabolites and proteins associated with anti-inflammatory and immunoregulatory functions (15). Preconditioning of cultured MSCs is now recognized as one approach to improving MSC functions *in vivo*. Hypoxia is recognized as an injury-related condition that when used to precondition MSCs leads to increased engraftment, angiogenesis and regenerative function (39). This study further underscores those factors such as TNFα in wounded tissues promote expression of MSC EVs with regenerative features. MSC preconditioning is considered an important approach to improving cultured MSC activity (40).

The use of TNFα preconditioning may have selected advantages over other methods of altering MSC-derived EV function. TNFα preconditioning does not appear to affect the general biophysical properties of the EVs which may account for their similar endocytosis when compared to naïve MSC EVs. One possible advantage of preconditioned MSC EVs is that preconditioning may be part of a cell culture strategy that minimizes variation from primary native MSCs. Another advantage of preconditioning of MSCs is that it increases the consistency of EV cargo as demonstrated at the protein and metabolite level as well as the miRNA level (41). Overexpression of a single miRNA affects a single miRNA function whereas preconditioning with cytokine or morphogen more comprehensively alters the miRNA content of the MSC EVs.

The present results indicate that MSC EVs treatment of the calvaria defect promotes bone regeneration by an indirect mechanism affecting macrophage polarization and that the resulting change in macrophage polarization precedes and influences subsequent and enhanced bone repair. Macrophage OSM expression was observed in these healing tissues and is indicative of one osteoinductive signal produced by macrophages that can be increased by macrophage exposure to TNFα EVs. Preliminary studies indicated that macrophages are essential for calvaria bone regeneration within these same collagen scaffolds. The reduction of monocytes using either clodronate liposome treatment in wild type mice or ablation of monocytes in MaFIA mice treated with AP20187 is associated with the marked reduction in calvaria bone regeneration (**Supplementary Figure 1**). This is consistent with reports of the role for osteomacs in fracture repair (42).

The control of macrophage plasticity ranging from M1 to M2 phenotypes is a critical process in the immunomodulatory control of bone regeneration (43). In the present investigation, TNFα EVs treatment of calvaria wounds was associated with a marked switch from an M1 predominant to an M2 predominant regenerative environment at the early times of healing. M1 macrophage-elaborated factors such as OSM assist in promoting osteogenesis (29, 43) and M2 macrophage-elaborated factors promote ongoing osseous regeneration (44).

When TNFα treated human gingiva derived MSC EVs were investigated, the preconditioning increased exosome number, induced M2 macrophage expression in a CD73-dependent manner and protected alveolar bone from ligature-induced periodontal bone loss. The TNFα preconditioning was associated with significant changes in the EVs miRNA cargo (38). Another study involving adipose stem cells demonstrated that exosomes isolated from cells preconditioned with TNFα promoted human primary osteoblastic cell proliferation and differentiation (45).

Our miRNA sequencing results show a significant change in the miRNA composition of the preconditioned MSC EVs. It is possible that a subset of these altered miRNA contributes to the enhanced immunomodulatory function. In preliminary analyses of some of the highly expressed miRNAs that are also differentially expressed, candidates such as miR-15b (46), miR-19b (47) and miR-22 (48, 49) stand out due to their established roles in immunomodulation. For example, miR-22 is a negative regulator of the NLRP3 inflammasome pathway (50) and has established anti-inflammatory function (50, 51). KEGG pathway analysis also indicated significant changes in pathways related to cancer, PI3K-Akt as well as the FoxO signaling pathways. Cancer related pathways are primarily immunomodulatory in nature as are the PI3K-Akt and the FoxO signaling cascades (52). These results indicate that the miRNA EV cargo shifts towards an immunomodulatory role when MSCs are preconditioned with TNFα. Ongoing studies continue to investigate more broadly the inflammation preconditioning of MSC EVs. Such studies are aimed at further defining the role of EVs and their miRNA cargo in the immunoregulatory effects of MSCs on tissue regeneration and repair.

We have observed an indirect effect of MSC EVs acting on macrophages to promote osteogenesis that may be complex in mechanism. Macrophages influence osteoblasts directly by secreted cytokines included OSM, BMP, and others. Here we demonstrate that MSC EVs treatment of calvaria resulted in the increased expression of OSM in healing tissues. Other effects cannot be excluded. For example, calvaria defect treatment with exosomes promoted neovascularization in support of osteogenesis (53). While the preconditioning of MSCs also influences cytokine expression that affects macrophage polarization, the isolation of EVs from conditioned media likely excludes cytokine effects in the present study; studies comparing EV depleted conditioned media with EVs demonstrate the effects of EVs on target cells is distinct from the EV depleted media (54). While TNFα EVs may interact with diverse cell types *in vivo*, the present cell culture studies discount a direct EV-mediated effect on osteoprogenitor cells in the calvaria.

In conclusion, the TNFα preconditioning of human MSCs results in EVs able to alter the macrophage phenotype *in vitro* and *in vivo*. While both control/naïve and TNFα EVs promote MSC osteoinduction at similar levels, the TNFα EVs increase bone regeneration in calvaria in a process that is accompanied by significant immunomodulation represented by an increase in the M2 macrophage population and suppression of M1 inflammatory cytokine production. Given the present observations, the TNFα preconditioned MSC EV’s promotion of bone regeneration is indirect and dependent on their immunomodulatory function in the regeneration process.

## Data Availability Statement

The datasets presented in this study can be found in online repositories. The names of the repository/repositories and accession number(s) can be found below: https://www.ncbi.nlm.nih.gov/, accession ID: PRJNA814986.

## Ethics Statement

The animal study was reviewed and approved by University of Illinois Chicago Animal Care Committee (UICAnimal Welfare Assurance No. A3460-01).

## Author Contributions

MK planned and executed the immunomodulatory components of this manuscript. C-CH planned and executed the bone regeneration components of this manuscript. SS performed *in vitro* experiments with respect to MSC differentiation and assisted in animal surgery. YL performed the EV characterization and some *in vitro* experiments. PG performed all the animal surgeries. SR and LC conceptualized, supervised and wrote the manuscript together. All authors contributed to the article and approved the submitted version.

## Funding

This study was supported by the National Institute of Dental and Craniofacial Research (R01DE027404, R01DE030495).

## Conflict of Interest

The authors declare that the research was conducted in the absence of any commercial or financial relationships that could be construed as a potential conflict of interest.

## Publisher’s Note

All claims expressed in this article are solely those of the authors and do not necessarily represent those of their affiliated organizations, or those of the publisher, the editors and the reviewers. Any product that may be evaluated in this article, or claim that may be made by its manufacturer, is not guaranteed or endorsed by the publisher.

## Supplementary Material

Supplementary Figure 1 Macrophage depletion leads to impair bone healing. **(A)** Normalized cell number of F4/80-CD11b double positive cells by flow cytometry analysis. To deplete macrophages, clodronate liposome (Liposoma, clodronateliposomes.com) was administrated *via* intraperitoneal injection as per manufacture’s recommended protocol up to 2 weeks. First administration was performed 3 days prior to ensure depletion of macrophages upon the surgery. The blood samples were collected retro-orbitally at day 4 and day 14 of clodronate liposome administration. *: statistical significance (P < 0.05) with respect to control calculated by Student’s t-test (n = 3). **(B)** Representative 3D μCT images of mouse calvarial defects at 8 weeks post wounding. Unilateral 3.5mm diameter defect was created using a trephine burr and recombinant BMP2 (500ng) soaked collagen scaffold was placed onto the defect. Note the impaired bone healing in clodronate group.
