# Supplementary figures and images for: Extracellular Vesicles From TNFα Preconditioned MSCs: Effects on Immunomodulation and Bone Regeneration
Source: Front Immunol. 2022 May 2;13:878194. doi: 10.3389/fimmu.2022.878194 (PMC9108364; doi:10.3389/fimmu.2022.878194)

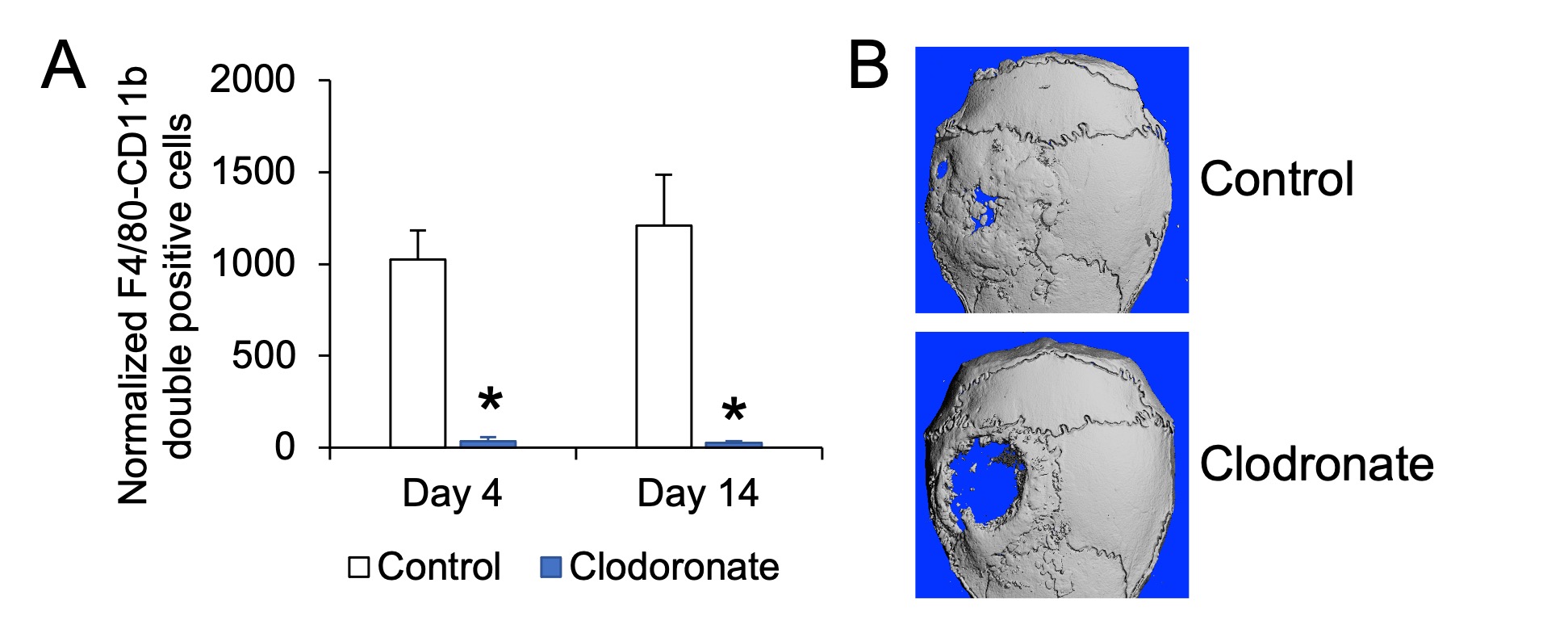

Supplement: Supplementary Figure 1 — Macrophage depletion leads to impair bone healing. (A) Normalized cell number of F4/80-CD11b double positive cells by flow cytometry analysis. To deplete macrophages, clodronate liposome (Liposoma, clodronateliposomes.com) was administrated via intraperitoneal injection as per manufacture’s recommended protocol up to 2 weeks. First administration was performed 3 days prior to ensure depletion of macrophages upon the surgery. The blood samples were collected retro-orbitally at day 4 and day 14 of clodronate liposome administration. *: statistical significance (P < 0.05) with respect to control calculated by Student’s t-test (n = 3). (B) Representative 3D μCT images of mouse calvarial defects at 8 weeks post wounding. Unilateral 3.5mm diameter defect was created using a trephine burr and recombinant BMP2 (500ng) soaked collagen scaffold was placed onto the defect. Note the impaired bone healing in clodronate group. [file Image_1.jpeg]
